# Tuberculosis patients’ knowledge and beliefs about tuberculosis: a mixed methods study from the Pacific Island nation of Vanuatu

**DOI:** 10.1186/1471-2458-14-467

**Published:** 2014-05-17

**Authors:** Kerri A Viney, Penelope Johnson, Markleen Tagaro, Saen Fanai, Nguyen N Linh, Paul Kelly, David Harley, Adrian Sleigh

**Affiliations:** 1 Secretariat of the Pacific Community, BP D5, 98848, Noumea Cedex, New Caledonia; 2 National Centre for Epidemiology and Population Health, Australian National University, Building 62, Corner of Eggleston and Mills Roads, 0200 Canberra, Australian Capital Territory, Australia; 3 History and Language (RSPAS), College of Asia and the Pacific, Australian National University. School of Culture, Canberra ACT 0200, Australia; 4 Ministry of Health, PB 9009 Port Vila, Vanuatu; 5 Global TB Programme, World Health Organization; formerly from The Division of Pacific Technical Support, World Health Organization Representative Office in the South Pacific, Suva, Fiji; 6 Population Health Division, ACT Health, ACT Government, GPO Box 825, Canberra City ACT 2601, Australia; 7 Australian National University Medical School, Level 2, Peter Baume Building, 42 Linnaeus Way, Canberra ACT 0200, Australia

**Keywords:** Tuberculosis, Vanuatu, Health-seeking, Diagnosis, Mixed-methods

## Abstract

**Background:**

The setting for this study was the Pacific island nation of Vanuatu, an archipelago of 82 islands, located in the South Pacific Ocean. Our objective was to assess the knowledge, attitudes and practices of tuberculosis (TB) patients towards TB.

**Methods:**

This was a descriptive study using qualitative and quantitative methods. Quantitative analysis was based on the responses provided to closed questions, and we present frequencies to describe the TB patients’ knowledge, attitudes and practice relating to TB. Qualitative analysis was based on open questions permitting fuller explanations. We used thematic analysis and developed *a posteriori* inductive categories to draw conclusions.

**Results:**

Thirty five TB patients were interviewed; 22 (63%) were male. They attributed TB to cigarettes, kava, alcohol, contaminated food, sharing eating utensils and “*kastom”* (the local term for the traditional way of life, but also for sorcery). Most (94%) did not attribute TB to a bacterial cause. However, almost all TB patients (89%) thought that TB was best treated at a hospital with antibiotics. Three quarters (74%) experienced stigma after their TB diagnosis.

Seeking health care from a traditional healer was common; 54% of TB patients stated that they would first consult a traditional healer for any illness. When seeking a diagnosis for signs and symptoms of TB, 34% first consulted a traditional healer. Patients cited cost, distance and beliefs about TB causation as reasons for first consulting a traditional healer or going to the hospital. Of the TB patients who consulted a traditional healer first, there was an average of two weeks delay before they consulted the health service. In some cases, however, the delay was up to six years.

**Conclusion:**

The majority of the TB patients interviewed did not attribute TB to a bacterial cause. Consulting a traditional healer for health care, including while seeking a diagnosis for TB symptoms, was common and may have delayed diagnosis. People require better information about TB to correct commonly held misperceptions about the disease. Traditional healers could also be engaged with the national TB programme, in order to refer people with signs and symptoms of TB to the nearest health service.

## Background

Tuberculosis (TB) is strongly linked to poverty, and a range of other social, environmental and biological determinants [1]. In the Pacific Islands region, TB remains a public health problem [2] and rates have not decreased significantly over the last ten years despite increased funding, better public health evidence about how to manage the disease and targeted public heath interventions designed to decrease the burden of TB [2]. This is especially true in the lower income countries in the Pacific and in sub-populations that are socioeconomically disadvantaged. In these populations, TB rates are stagnating [2,3].

The Pacific Islands region comprises 22 Pacific Island countries and territories with varying levels of income and development, and with different health systems. The highest rates of TB occur in Kiribati, Marshall Islands, Papua New Guinea and Tuvalu [2]. In many of these communities, a generalised TB epidemic is taking place. In the Pacific communities affected by TB, knowledge, attitudes and behaviours about TB can determine health-seeking behaviour, adherence to TB treatment, TB treatment outcomes and ongoing transmission of TB.

TB patients’ health-seeking behaviour informs national TB programmes (NTPs) on improved case detection and how diagnostic and treatment delays can be reduced. Most available data comes from high burden countries, particularly in Africa, but also India and Asia [4-8]. One study from the Pacific explored the reasons for late presentation to hospital following onset of TB symptoms [9]. However, there is little other research on TB knowledge, attitudes, and behaviours (in particular health-seeking practices) in the Pacific, apart from information in some Demographic and Health Surveys (DHSs), that include a component on TB.

In studies conducted in Africa, TB patients believed that TB is caused by supernatural and physical causes [4,10,11]. In rural Malawi TB was believed to be sexually transmitted [12] and in Kenya, TB patients attributed it to smoking, alcohol, hard work, exposure to the cold, hereditary factors, and exposure to other TB patients [10]. In rural Uganda, TB patients, traditional healers and community leaders attributed TB to shared use of cooking and eating utensils, heavy labour, smoking, bewitchment and hereditary factors [4]. In rural South Africa, TB patients and the community attributed TB to breaking cultural rules that demand abstinence from sex, environmental pollution, smoking and alcohol [11], while in Mwanza, Tanzania, TB patients attributed TB to similar factors; smoking, alcohol, bewitchment, and hereditary factors [13]. In a study of Filipino immigrants in the United States, causal beliefs included exposure to bacteria and viruses, smoking, alcohol, overwork, poor nutrition, colds, cough and fever, and contact with a person with TB [14].

Most studies and surveys are predicated on the medical explanation of TB. For example, in the DHSs in the Pacific Islands region, respondents are often asked: whether they have heard of TB, whether it is spread through the air, if it can be cured, and if they would keep a TB diagnosis secret. In the Kiribati DHS, 81% of women and 77% of men who had heard about TB stated that it was spread through the air [15]. In Solomon Islands, a slightly larger proportion of men (86%) than women (82%) reported that TB is spread through the air by coughing [16], and in Tuvalu, 61% of women and 51% of men who had heard of TB reported that it spreads through the air by coughing or sneezing [17].

Worldwide, many people with symptoms of TB consult traditional healers, often before seeking western medical care [5,18,19]. The proportion of people who would first consult a traditional healer depends on the setting, and on contextual factors, including cost, ease of access and local beliefs about TB causation [19-22]. In studies from three countries in Africa, between 6% and 51% of TB patients first consulted a traditional healer [5,23-25]. Aetiological beliefs strongly influence choice of care, and if witchcraft is considered the cause, a traditional healer will likely be sought first [4,9,11]. Some patients consult traditional healers western medical services concurrently [4,19]. TB patients may also opt to self-treat with home remedies or over-the-counter medications [26,27].

Knowledge, attitudes and behaviour of TB patients have not been reported for the Pacific in the peer-reviewed scientific literature. Indigenous staff working for NTPs in Pacific Islands, particularly in Melanesia, report that: a) consultation with a traditional healer as part of TB health-seeking behaviour is common; b) both western medicine and traditional medicine are often taken concurrently by people with TB; c) consultation with traditional healers may delay TB diagnosis; and d) collaboration between the Ministry of Health and traditional healers may be possible but requires further research.

In Vanuatu – a Melanesian country in the Pacific Ocean – the NTP staff noted that many of their TB patients reported accessing traditional healers before attending the outpatient clinic. Further, they reported that many ni-Vanuatu TB patients have advanced disease at hospital presentation, perhaps caused by of a delay in TB diagnosis involving traditional medicine and care. Therefore, we decided to assess the knowledge, attitudes and self-reported behaviours of TB patients in Vanuatu. Our specific objectives were to: a) describe the characteristics of a group of TB patients from four sites in Vanuatu; b) describe knowledge about TB among these patients; and c) describe their health-seeking practices and behaviour.

## Methods

### Study design

This study used a combination of qualitative and quantitative methods. We used ethnographic methods, recruiting indigenous researchers to conduct individual interviews that documented the knowledge, attitudes and behaviours of TB patients. Semi-structured questionnaires containing both closed and open-ended questions were used to determine knowledge, attitudes and practices regarding the cause, diagnosis, health-seeking behaviour and treatment of TB. Our analysis used a grounded-theory approach, where interview data were organised thematically, creating various unique categories, which in turn were used to explain and create a theoretical perspective [28].Our study adhered to the RATS guidelines for qualitative research (i.e. Relevance, Appropriateness, Transparency and Soundness of interpretive approach) [29].

### Setting

**Figure 1:**
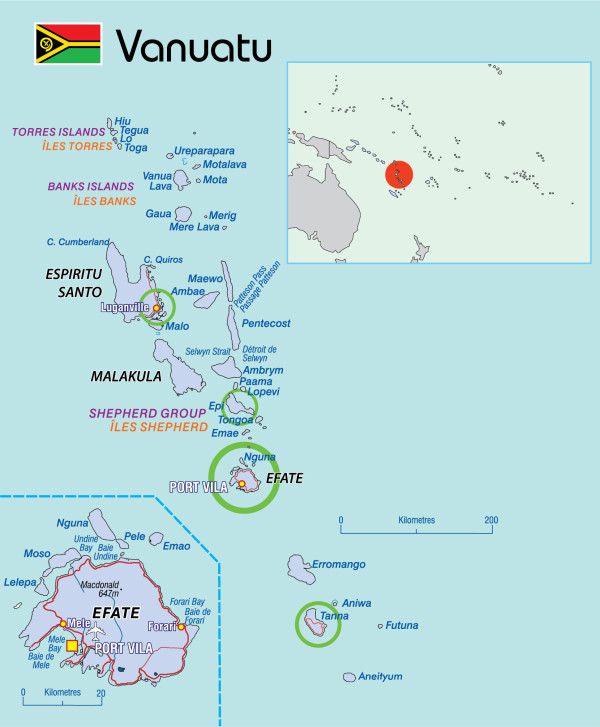
**Map of Vanuatu, showing the four study sites (circled in green) (see separate file).** Source: Secretariat of the Pacific Community (permission granted for reproduction).

The study was conducted in the Republic of Vanuatu. Vanuatu is an island nation in the South Pacific, located approximately 1,750 kilometres east of northern Australia [30]. It is an archipelago of 82 islands, of which 65 are inhabited, and the country is divided into six administrative provinces (Tafea, Shefa, Malampa, Penama, Sanma and Torba) (Figure 1) [30]. The population of approximately 260,000 people are 98.5% ni-Vanuatu, of Melanesian descent [30]. Eighty percent of the population live in rural areas where subsistence agriculture provides the main source of income [30]. The national language of Vanuatu is Bislama, a pidgin English [30].

### Health care in Vanuatu, and the role of “kastom”

Health care in Vanuatu is provided by the national government (the Ministry of Health) with little involvement of the private sector. There are two regional referral hospitals, three provincial hospitals, 30 health centres, 97 dispensaries and 231 aid posts located throughout the country, making primary health care accessible to most of the population [31]. After independence in 1980, the government introduced a fee-based system for inpatient and outpatient care [31]. Free health care was then introduced in 1993 [31]. However, patients may still have to pay a contribution fee for outpatient services [31].

The population of the Vanuatu archipelago includes people with a wide range of social systems and customs. “*Kastom”*, a local word that is derived from the English word custom, is used to refer to traditional ways of life and includes religion, art, economics and magic. In some situations, the word “*kastom”* is associated with sorcery [32], but standard usage refers to the ni-Vanuatu way of life, observance and tradition.

In addition, many traditional elements of life in Vanuatu are influenced by Christianity, introduced by missionaries in the 19th century [30].

The introduction of Christianity into Vanuatu did not totally eradicate traditional beliefs and practices but resulted in a synchronistic system, where the new ways of life “implied some measure of willing cooperation on the part of local people” [33]. In parts of Vanuatu where people hold traditional beliefs, social taboos associated with the notion of pollution - usually by blood during menstruation and childbirth, food preparation and sexual intercourse - are common. Such taboos may align with fear of deliberate poisoning or malevolent magic.

Traditional healers or “*klevas”* are accessible throughout Vanuatu [34,35]. Their practice is termed “*kastom”* medicine and is based on traditional practices and beliefs [35,36]. “*Klevas” -* who are usually male- are believed to receive their healing powers from their ancestors and they are widely used by the community throughout Vanuatu [35,37]. Traditional healers in Vanuatu are not enumerated, but they are accessible in almost all villages, and there may be as many as 1,000 located throughout the country [38]. When unwell, most ni-Vanuatu people will access health care from a traditional healer before seeking health care in the government funded health care system [37]. After doing so, they will be offered leaf medicine, prayer, water treatments, massage or bone-setting [34,35]. Another important cultural feature in Vanuatu is the village “*nakamal”*, or meeting house, in which kava – a mildly intoxicating drink found across the Pacific made from the roots of the plant *Piper methysticum* – is consumed [39]. Traditionally, kava was prepared by chewing the roots before adding water to them, and it is still prepared in this way in some areas of Vanuatu [39].

Vanuatu reports approximately 120 cases of TB each year and is regarded as a medium burden TB country in the Pacific [3,40]. The notification rate of TB has decreased in the past twenty years, from 95/100,000 population in 1990 to 48/100,000 population in 2010 [3]. However, in the period 2005 to 2010 the incidence of TB has not declined [3]. The Vanuatu Ministry of Health funds a dedicated NTP, which is well managed by nursing staff trained in the programmatic management of TB. The World Health Organization (WHO) estimates that 70-80% of all TB cases in Vanuatu are detected, diagnosed and treated. Vanuatu reports good treatment success rates; an indicator of programmatic success [3].However, an estimated 20-30% of incident TB cases remain undiagnosed so there may be barriers to accessing TB care. Anecdotally, many TB patients access traditional medicine before seeking health care from an aid post or health centre

### Participants and sampling

Participants in our study were TB patients aged 18 years or older who were registered in the national TB register from September 2010 to February 2012 from one of four study sites: 1) Port Vila (the capital of Vanuatu on Efate island in Shefa Province), 2) Luganville (Vanuatu’s second largest city and the provincial capital of Sanma Province, in Santo), 3) Tanna (the most populous island and provincial capital of Tafea Province) and 4) the island of Epi (an island in the Shepherd Islands group in Shefa Province). In the last five years 71% of Vanuatu TB patients have come from the four study sites. Participants included the patients who were currently on TB treatment, and patients who had recently completed TB treatment at the time of the study.

To enrol patients in the study, the interviewers reviewed the national TB register and identified TB patients from the four study sites who were registered during the study period. The national TB register is a document accessible to all NTP staff, containing all TB patients who have been diagnosed with TB by a physician, who are receiving treatment for TB and who have been notified as a case of TB to the NTP. Eligible patients were approached at the TB clinic, hospital or at home to invite participation in the study. If patients were not available upon first visit (i.e. not at home or not in their hospital bed) they were not revisited. The same approach was used for patients who were currently on or had completed TB treatment. Participants were interviewed between October 2011 and February 2012 and were recruited until thematic saturation was obtained. No material incentives were provided. All participants provided written informed consent to participate, and all consented to having the interview recorded.

### Data collection

We developed a structured questionnaire for our study which contained open and closed questions and interviewers were able to ask additional questions if further clarification was needed. All participants were interviewed by one of five trained nurse interviewers and the interviews were conducted in either Bislama, or for participants in Tanna, in east-Tannese, one of the local languages. The interviewers were all local nurses who were working for the NTP at the time of the interviews. All interviews were recorded, and abbreviated responses were written on the paper data collection forms in either Bislama or English. The recorded interviews were transcribed in Bislama, then translated into English. The English translations of the questionnaires were used for the analysis. The participants did not verify the content of their responses after transcription and translation, for logistical reasons.

The questionnaire was divided into two sections. Section A contained questions about patient demographics and general knowledge about different illnesses in the community, including TB. Section B sought information about the patients’ health-seeking practices, use of traditional healers and information about the patients’ TB diagnosis.

### Analysis

#### Quantitative analysis

Quantitative data were double entered into EpiData version 3.1, and were verified and corrected prior to analysis. Descriptive analyses were carried out in EpiData Analysis version 2.2.2.178. Quantitative analysis was based on the responses provided to closed questions.

#### Qualitative analysis

Qualitative analysis was based on the responses provided to open questions. The translated questionnaires were reviewed in detail by three members of the research team (PJ, MT and KV) and discussions were held to better understand the data and explore key categories and themes. Thematic analysis was used; the interview texts were coded, followed by grouping of the codes into categories and themes. Content analysis was carried out manually by two members of the research group (PJ and KV). Discrepancies were resolved by consensus.

### Ethical considerations

Ethics approval was obtained from the WHO (Western Pacific Region Office) Ethics Review Committee and the Australian National University Human Research Ethics Committee. Vanuatu does not have a local health ethics committee, however, the Vanuatu Ministry of Health and the Vanuatu National Cultural Council gave permission to conduct the study. The research was carried out in accordance with the principles of the Vanuatu Cultural Research Policy [41].

Written and oral information about the nature and objectives of the study was provided to participants prior to written informed consent. The data collection forms, interview questionnaires and transcripts were stored in a secure and locked cupboard at the Secretariat of the Pacific Community office and were only accessible by the study investigators.

## Results

### Demographic and disease characteristics

A total of 35 TB patients were interviewed. One TB patient was approached for interview, but declined. The demographic and disease characteristics of the patients are described in Table 1. Ten patients were enrolled from Port Vila, ten each from Tanna and Santo, and five from Epi. Almost two thirds (22, 63%) of the patients were male and 16 (46%) were aged between 35 and 54. Most of the patients had pulmonary TB (30, 86%). Fifty-four percent (19) were currently on TB treatment.

**Table 1:** Demographic and disease characteristics of 35 TB patients in Vanuatu: 2011-2012

**Characteristic (n = 35)**	**Number**	**Percentage**
Age group		
18-34 years	12	34
35-54 years	16	46
55 years and over	7	20
Gender		
Female	13	37
Male	22	63
Location		
Epi	5	14
Port Vila	10	29
Santo	10	29
Tanna	10	29
Type of TB		
Extra-pulmonary	5	14
Pulmonary smear negative	8	23
Pulmonary smear positive	22	63
Current or past TB		
Current TB	19	54
Past TB	16	46

Participants described their local communities as places where there were a lot of different types of illnesses, ranging from communicable diseases such as TB and gonorrhoea to non-communicable diseases such as diabetes and cancer. Many of the participants described a range of symptoms (as opposed to distinct disease entities) that people suffered from in their communities.

### TB symptoms

Participants described a variety of symptoms that are typically associated with TB (such as cough, fever, loss of appetite, weight loss, night sweats, and shortness of breath) and a variety of non-specific symptoms. Cough, fever and weight loss were the most frequently reported symptoms.

### Causes of TB

The causes of TB described by the patients are outlined in Table 2. Most patients were not aware that TB was caused by a bacterium (33, 94%). Many participants attributed their TB to smoking, and alcohol or kava preparation and consumption:

**Table 2:** Beliefs about TB causation as described by 35 TB patients in Vanuatu: 2011-2012

Description of cause*	**Number**	**Percentage**
Smoking	9	26
Food^	7	20
Kava	6	17
Alcohol	5	14
Hard work	4	11
*Kastom*	4	11
Poor nutrition	3	9
Coughing	2	6
Bacteria^#^	2	6
Sharing eating utensils	2	6
Stress and worry	2	6
Hereditary	2	6
Food prepared by a menstruating woman	1	3
Inadequate rest	1	3
Marijuana	1	3
Weakness	1	3
Skin infection	1	3
Accident followed by blood clots	1	3

*The TB patients described more than one cause of TB.

Therefore these percentages do not add up to 100.

^Two responses in the food category were about eating food that may have been forbidden or poisoned. Therefore these responses could also be included under *kastom*.

^#^These responses indicated that the bacteria were either present in meat or feed off blood.

*“It starts if you start drinking a lot of kava and alcohol and even smoking.”* (Male TB patient, aged 43, TB3)

*“I think I got it from the nakamal* [traditional meeting place in Vanuatu] *by using the kava cups and sharing cigarettes.”* (Male TB patient, aged 56, TB14) [42]

Food and utensils were a recurring theme and many of the participants thought that their TB was caused by either food or the sharing of eating utensils:

*“I think it started from when we used to live with our grandparents and shared food and eating utensils and this is how the sickness was passed on.”* (Female TB patient, aged 23, TB7)

“Bad” food, not enough food or sharing plates with animals were also considered as causes. It was also thought that TB could be contracted through poisoned food:

*“..someone poisoned [cursed] my food with a kastom leaf and this poison was inside my body, that’s why they can’t get it out of me.”* (Male TB patient, aged 30, TB31)

Two participants stated that bacteria in food were the cause of their disease. Beliefs about TB causation also extended to stress, overwork, not getting enough rest, hereditary causes and “*kastom”*:

*“A person who usually practice[s] kastom rituals not following his regulations or rules of the specific kastom will be sick of TB.”* (Male TB patient, aged 46, TB2)

A quarter (9,26%) were not aware of local beliefs regarding TB causation but some considered smoking (11,31%), food and eating utensils (7,20%), kava and alcohol consumption (3,9%) and *kastom* (2,6%) to be the local beliefs about TB causation:

*“Yes, I have heard that belief sharing of food and eating utensils may also spread the disease. But things like chewing of kava make it more badly especially our preparation of kava on Tanna.”* (Male TB patient aged 60, TB4)

### Who gets TB

Just over half (18, 51%) of the participants thought that anyone could contract TB. Others thought that TB affected elderly people (9, 26%), children (3, 9%) and adults (1, 3%). Mothers, people who smoke, and people who work too hard were also considered at risk of developing TB by a few.

### Progression of TB

Just over half were not aware of what might make TB worse (18, 51%), although five people (14%) mentioned that not taking their medicine as prescribed would worsen their disease:

*“The dangerous thing about TB is that if you don’t take your medication as instructed you could get sicker. This guy from Malakula, he ran away during his treatment, he felt better and decided to go home but he hadn’t finished his treatment. If you go home and feel worse then come back to the hospital for treatment, what will you say to the nurses?”* (Male TB patient, aged 46, TB15)

Other factors thought to worsen TB disease were cold, poor nutrition, hard work, kava consumption and smoking.

### TB treatment

Nearly all of the participants (31, 89%) thought that TB was best treated in the hospital. Almost all participants (31, 89%) believed that TB was curable. When asked about the best treatment for TB, 31 (89%) concluded that western medicine was the best treatment and another four thought that concurrent western and traditional medicine would be the ideal treatment for TB:

*“I think prayer and the hospital [medicine] but kastom medicine cannot kill the TB bacteria, it can relieve cough but it can’t kill the TB bacteria.*” (Male TB patient aged 24, TB13)

*“Western and kastom medicine can help cure TB.”* (Male TB patient aged 30, TB31)

Respondents said that the most harmful thing about TB was that it could kill (16, 46%) while other effects of TB considered harmful were transmissibility, the requirement for medication that must be taken continuously, and stigma. When asked if TB can kill, 31 (89%) said that they believed that it could kill or they had heard about someone who had died from TB.

### Stigma

Twenty-six (74%) said that they felt that they were treated differently by other people after being diagnosed with TB, resulting in others avoiding or refusing to eat with them. The participants felt that people avoided them due to concerns about TB transmission. Almost half (16, 46%) said that TB treatment lessened the stigma, but 11 (31%) people said that TB-related stigma does not change:

*“Communities in Vanuatu are afraid of people who have TB. At first we didn’t have diabetes, AIDS and cancer, not even TB, but today they say TB is the most dangerous. People tend to avoid those who have TB because they are scared of catching TB. But if there is awareness about TB people will know and be proud that TB can be treated.”* (Female TB patient, aged 37, TB16)

### General health-seeking behaviour

The participants were asked a range of questions about their general health-seeking behaviour. To put this into context they were also asked about their perception of the usual health-seeking practices in their community.

Over half of the participants (19, 54%) reported that they would usually seek health care from a traditional healer when seeking medical care for any kind of illness, (Table 3), and the participants routinely said that they would seek care from a traditional healer in the first instance followed by a visit to the local aid post or hospital if required (i.e. if symptoms were not resolving or if traditional medicine ceased to be effective).

**Table 3:** Reported health-seeking practices of 35 TB patients in Vanuatu: 2011-2012

**Health-seeking practice (n = 35)**	**Number**	**Percentage**
Who does the patient consult first?		
Health service	14	40
Traditional healer (*Kleva*)*	19	54
Depends	2	6
Who do villagers consult first?		
Health service	3	9
Traditional healer (*Kleva*)	21	60
Depends	11	31
Is cost a factor?		
Yes	17	49
No	5	14
Not stated	13	37

*Kleva: Bislama word for a traditional healer or traditional medical practitioner.

*“Like I said people don’t go to the hospital first, only when the traditional healer’s medicine is no longer helping that we think of referring to the hospital. They don’t usually go to the hospital first, only pray and leaf medicine.”* (Male TB patient aged 60, TB4)

*“If what I have is a kastom sickness then I go to the traditional healer. But when it’s a hospital sickness, I go to the hospital for fever, cough, TB and some others as well.”* (Male TB patient aged 46, TB15)

When participants were asked about the health-seeking behaviour of people in their community they said that most people (21, 60%) would visit a traditional healer first, however 11 people thought that it would depend on the type of illness and the person’s individual circumstances (Table 2).

*“Everyone has their own opinion, if they want to go to the traditional healer first, then they go, if they want to go to hospital first, then they go. In our village almost everyone goes to a traditional healer first.”* (Male TB patient aged 43, TB11)

*“I go to a traditional healer because if I go to see him he’ll make me better but if it doesn’t work I’ll go to the hospital.”* (Female TB patient aged 53, TB18)

Many (13, 37%) did not answer questions on the cost of hospital and traditional treatment but 17 (49%) agreed that cost was a factor and many also talked about the distance they needed to travel to get to a hospital (which related to cost of fuel and transport, as well as the time needed to travel to a hospital) and the convenience of seeing a traditional healer who is often close by:

*“Cost might be a reason because the hospital is far from where we are so to get transport to a hospital it’s 6000 Vatu [63USD] and when some can’t afford it they go to a traditional healer.”* (Male TB patient aged 58, TB32)

*“…distance is too far and [it is] expensive to pay for transport and also paying the outpatient department fee.”* (Male TB patient aged 36, TB6)

However, others thought that a person’s belief about illness causation and cure rather than cost was the factor that determined where people first sought care when sick:

*“I don’t think that the hospital cost is high, it comes down to people’s beliefs, they believe that if they go to a traditional healer they’ll be cured when that doesn’t work, go well then they go to the hospital and realise that they should have gone to the hospital in the first place. It’s just a strong belief that they have.”* (Male TB patient aged 56, TB25)

### Health-seeking behaviour for *short wind* and TB symptoms

Participants were asked a series of questions about “*short wind”* (a local term to describe lung, chest or breathing illnesses) and their own pathways to a TB diagnosis. “*Short wind”* was most often interpreted by the participants as asthma, or sometimes influenza.

When asked if people would consult a healer for “*short wind”*, almost half of the respondents said that a healer would not be consulted (17, 49%). Forty percent of respondents stated that people with “*short wind”* may self-refer to a hospital after they had tried “*kastom”* medicine when it had provided no relief from symptoms.

**Figure 2:**
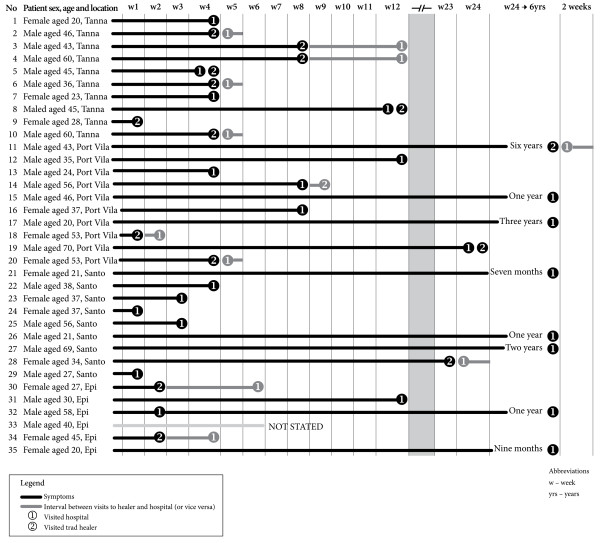
Time to diagnosis and type of health care accessed for 35 TB patients in Vanuatu (see separate file).

When seeking a diagnosis for signs and symptoms of TB, 20 (57%) first consulted the hospital and 12 (34%) a healer. The mean time between consulting a healer and the hospital was 1.9 weeks (or 13 days) for the 12 who first consulted a healer (Figure 2). However, there were eight patients who described having TB symptoms for more than six months before they were diagnosed (the range of duration of TB symptoms in these patients was seven months–six years) and for seven of these people it was not clear when they had seen a traditional healer during their symptomatic period (Figure 2).

Participants were asked how traditional healers treat TB and many of the patients did not know or did not provide specific details of this treatment, other than to say that the healer provided leaf or “*kastom”* medicines:

*“The traditional healer I went to prepared a leaf medicine for me and I drank it.”* (Male TB patient aged 56, TB14)

*“…women would be given the leaf juice while the men chew the leaf.”* (Female TB patient aged 20, TB35)

Participants accurately described hospital treatment and felt for the most part that the treatment was working well. However, a minority of patients said that they thought the treatment was working slowly. One patient stated:

*“Yes, we drink medicine and [do] I think is it working slow or fast? The medicine works slow but in the end it will kill the TB bacteria.”* (Male TB patient aged 69, TB27)

In addition, it was clear that a small number of patients (i.e. five) had initially been provided with inappropriate treatment including oral rehydration solution, paracetamol, and antibiotics that are not effective in curing TB (i.e. amoxycillin).

The impacts of TB were mainly physical in nature and related to the effects of TB symptoms, neglect of regular duties (including work, looking after gardens, looking after family and attending school), isolation from family due to hospitalisation and the effects of TB related stigma:

*“The main problem I had with TB was that my lungs hurt and I had shortness of breath.”* (Male TB patient aged 46, TB15)

*“When I was sick, I didn’t go to the nakamal, meetings and church because of my cough.”* (Female TB patient aged 27, TB30)

Participants were asked what they thought was most feared about TB. The fact that TB is a transmissible disease was something to fear, as was the stigma that relates to TB and the potentially fatal nature of TB:

*“People think that TB is dangerous because they fear that they may catch it and financially they wouldn’t be able to afford it.”* (Female TB patient aged 44, TB34)

*“That people will be afraid of them, that people and families would not like them to sleep, share food and share spoons and forks and plates…That society, people and family will not accept him as a family [member] and friend anymore. That they will lose friends and will lose the support from their families.”* (Male TB patient aged 45, TB8)

Most participants were hopeful that their lives would improve once their TB was treated and cured. The participants noted that they would be able to resume their regular duties (such as working, gardening, looking after children and going to school), reassess their workload to reduce the likelihood of overworking and experience an alleviation of their TB symptoms. In addition, many of the respondents stated that they would change behaviours by reducing their alcohol and kava intake, quitting cigarette and cannabis smoking, and modifying other behaviours that are perceived to be anti-social (i.e. promiscuity, stealing):

*“I know that my life will change now. I’m quitting kava, cigarettes and alcohol.”* (Male TB patient aged 56, TB14)

*“I change from going to see different women, having sex all about, working so hard and stealing. Now I have stopped.”* (Male TB patient aged 20, TB17)

## Discussion

### TB symptoms and causation

We attempted to better understand the knowledge, attitudes and behaviours of a group of TB patients in Vanuatu. The patients were aware of TB and other diseases in their communities and could describe their symptoms. They were generally not aware of the bacterial cause of TB and thought that TB could be caused by a range of factors including shared food and eating utensils, kava and alcohol, cigarettes, hereditary causes and “*kastom”*. These findings are consistent with studies from many African countries in which beliefs about TB causation include witchcraft, smoking, sharing eating utensils, and heavy labour [4,10,43]. An incorrect understanding of TB causation can influence patients’ health-seeking behaviour, adherence to a prescribed treatment regimen and treatment outcome [7,11,24,27,44]. A misunderstanding of the microbial cause of TB can also lead to delays in diagnosis in resource limited settings [18,23,45,46]. Further, the initial symptoms of TB may resemble other diseases (i.e. fever for malaria, cough for other lung diseases) therefore it is possible that the non-specific, insidious nature of some TB symptoms leads to diagnostic delay. First consultation with a traditional healer may also result in delays to TB diagnosis [24], as these subtle differences between TB and other diseases may not be distinguished, and traditional healers do not have access to TB diagnostic services.

### Stigma

Three quarters of our study patients experienced and were troubled by stigmatisation. Despite national and international efforts to lessen TB related stigma, patients described feelings of fear, shame and embarrassment when diagnosed with TB [11,18,47,48]. Stigmatisation was mainly limited to avoidance of TB patients (due to fear of transmission) and refusing to share food and eating utensils. Stigmatisation may lead to diagnostic delay and ongoing transmission of TB in the community [10]. We were not able to confirm this definitively in our study, but it is likely that some delays in seeking a diagnosis may have been related to the stigma associated with a diagnosis of TB.

### Consultation with traditional healers

Approximately one third of participants reported that they first consulted a traditional healer when seeking a TB diagnosis. In communities where there is a strong tradition of traditional healers and spiritual causation of illness, seeking health care from traditional healers is the norm. In studies of TB patients’ health-seeking behaviour in other low income countries, consultation with a traditional healer as the first point of TB care is commonly reported [7,10,22]. In rural South Africa one quarter to one half consulted a traditional healer first [11,24]. Likewise, in Tanzania, The Gambia, Kenya and Malawi, consultation with a traditional healer prior to going to hospital was common [7,10,22-24,26,49]. Results similar to ours have been obtained in South Africa and Malawi, with approximately one quarter of patients first consulting a traditional healer [11,49]. It is desirable that patients with presumptive TB should first consult their local hospital instead of a traditional healer so that a definitive diagnosis can be made and the correct combination of antibiotics can be prescribed. However, changing beliefs and behaviours is a complex task, which may be best managed by local chiefs, church leaders, the community members and leaders, and traditional healers themselves.

In our study, some patients visited a traditional healer due to the lower cost of doing so or the geographical proximity of the healer. There are approximately 200 known traditional healers in Vanuatu (and possibly up to 1,000) [38]; each village has one or more traditional healers, which means they are very accessible. Many do not demand payment for their services and are given a token of appreciation (which may not be monetary) by their patients. In Vanuatu western health care is available across the nation but in some cases access is difficult [31]. The cost of accessing outpatient services in our study sites is approximately 200 Vatu (for adults) and 100 Vatu for children (“*pikinini”*), equating to USD 2.20 and USD 1.10 respectively (Current Gross Domestic Product is $3037 USD per capita) [50]. The cost of transport may also deter people from going to the hospital, as the price of fuel (for cars and boats) and airfares (if required) are relatively expensive, and beyond the reach of many ni-Vanuatu. Cost and distance to hospital are factors that may delay diagnosis and lead to consultation with a traditional healer [22,45,46,51-53]. Borrowing money for a hospital visit was associated with long patient delays in rural, mountainous Thailand (>21 days) [8]. Distance to a health facility may also delay diagnosis; in another study which assessed health-seeking and utilisation of traditional healers in Zambia, 49% of patients could walk to see a traditional healer in less than 30 minutes while the hospital was the same distance for 34% [21].

Seeking care from the hospital and a healer concurrently was reported infrequently in our study. In other settings, however, seeking treatment from the hospital system and traditional healers is common [4,19,54].

### Limitations

The study used a convenience sample. Therefore, the findings may not be generalisable to all of Vanuatu. However, we gained insights into what TB patients believe about TB, in order to assess some of their knowledge about TB and better understand their self-reported health-seeking behaviours. Many of those interviewed had successfully completed TB treatment and therefore may have had a positive experience with western TB treatment. We were not able to interview people who were unable to access this treatment (people whose TB had never been diagnosed) or members of the general community. However, this study was complemented by a study on traditional healers to assess their knowledge, attitudes and self-reported behaviours towards TB. Bias may have been introduced by the type of interviewer chosen. All interviewers were TB nurses employed by the Ministry of Health, and participants may have wanted to please the TB nurses with their answers. However, it was explained that participation in this study would not affect TB treatment or the attitudes of the TB staff towards them, the response rate was very high and many reported contact with traditional healers. In addition, our findings were very similar to other studies, suggesting bias was not significant.

## Conclusions

This study highlights some of the challenges in delivering TB care in Vanuatu. The majority of TB patients interviewed did not attribute TB to a bacterial cause. Many patients experienced TB related stigma and frequently consulted a traditional healer. Long diagnostic delays were reported by almost a quarter of the patients. Improved education for communities about TB signs and symptoms and where to seek care for signs and symptoms of TB may reduce these delays. Engaging traditional healers may have the added benefit of strengthening the links between western and traditional medicine in Vanuatu and may be helpful for people with TB and other health conditions. Our study provides new evidence on TB knowledge and involvement of traditional healers in TB management in Vanuatu, which can potentially be used to enhance TB services and to better engage traditional healers in TB management.

## Abbreviations

DHS: Demographic and health survey; KV: Kerri Viney; MT: Markleen Tagaro; NTP: National tuberculosis programme; PJ: Penelope Johnson; TB: Tuberculosis; USD: United States Dollars; WHO: World Health Organization.

## Competing interests

The authors declare that they have no competing interests.

## Authors’ contributions

KV conceived and designed the study, analysed the data and drafted the first version of the paper. PJ designed the study, advised on data analysis and interpretation and drafted the manuscript. NNL assisted with the study design, provided input into data interpretation and revised the manuscript. MT conceived the concept of the study, assisted with the study design, co-ordinated data collection, and assisted with analysis and interpretation of the data, and revising the manuscript. SF collected data and assisted with data interpretation and revising the manuscript. AS provided advice on data analysis and interpretation and revised the manuscript. DH provided advice on data analysis and interpretation, and revised the manuscript. PK also provided advice on study design, assisted with the funding and ethics applications, provided advice on data analysis and interpretation and revised the manuscript. All authors read and approved the final manuscript.

## Pre-publication history

The pre-publication history for this paper can be accessed here:

http://www.biomedcentral.com/1471-2458/14/467/prepub
